# An N-Shaped Beam Symmetrical Vibration Energy Harvester for Structural Health Monitoring of Aviation Pipelines

**DOI:** 10.3390/mi16080858

**Published:** 2025-07-25

**Authors:** Xutao Lu, Yingwei Qin, Zihao Jiang, Jing Li

**Affiliations:** 1 College of Mechatronics Engineering, North University of China, Taiyuan 030051, China; 2 School of Information and Communication Engineering, North University of China, Taiyuan 030051, China; 3 State Key Laboratory of Mechanics and Control for Aerospace Structures, Nanjing University of Aeronautics and Astronautics, Nanjing 210012, China; 2201013@nuaa.edu.cn; 4 School of Electrical and Control Engineering, North University of China, Taiyuan 030051, China; lijingnuc@163.com

**Keywords:** vibration energy harvesting, aviation pipelines, structural health monitoring

## Abstract

Wireless sensor networks provide a solution for structural health monitoring of aviation pipelines. In the installation environment of aviation pipelines, widespread vibrations can be utilized to extract energy through vibration energy harvesting technology to achieve self-powering of sensors. This study analyzed the vibration characteristics of aviation pipeline structures. The vibration characteristics and influencing factors of typical aviation pipeline structures were obtained through simulations and experiments. An N-shaped symmetric vibration energy harvester was designed considering the limited space in aviation pipeline structures. To improve the efficiency of electrical energy extraction from the vibration energy harvester, expand its operating frequency band, and achieve efficient vibration energy harvesting, this study first analyzed its natural frequency characteristics through theoretical analysis. Finite element simulation software was then used to analyze the effects of the external excitation acceleration direction, mass and combination of counterweights, piezoelectric sheet length, and piezoelectric material placement on the output power of the energy harvester. The structural parameters of the vibration energy harvester were optimized, and the optimal working conditions were determined. The experimental results indicate that the N-shaped symmetric vibration energy harvester designed and optimized in this study improves the efficiency of vibration energy harvesting and can be arranged in the limited space of aviation pipeline structures. It achieves efficient energy harvesting under multi-modal conditions, different excitation directions, and a wide operating frequency band, thus meeting the practical application requirement and engineering feasibility of aircraft design.

## 1. Introduction

With the continuous advancement of aviation technology, the application of aircraft in various extreme environments is becoming increasingly widespread [1]. As a crucial component of aircraft, aviation pipeline systems are responsible for transmitting various media such as fuel, hydraulic oil, and coolant. The operational status of these systems is directly related to the safety and reliability of the aircraft [2]. Traditional aviation pipeline monitoring methods primarily rely on periodic inspections and maintenance [3]. However, these methods are time-consuming, labor-intensive, and have a significant risk of missed detections, making real-time monitoring of the pipeline’s operational status difficult [4]. Therefore, developing an efficient and real-time aviation pipeline monitoring system has become an urgent issue to address. Wireless sensor networks (WSN), as an emerging monitoring technology, offer advantages such as flexible deployment and convenient data transmission and have been widely applied in various monitoring scenarios [5,6]. However, the complex working environment of aviation pipeline systems presents a challenge for the power supply of sensor nodes, which has been a key factor limiting the application of WSN [7,8]. Traditional battery-powered methods have limitations such as limited lifespan and the need for regular replacement, making them unsuitable for long-term, unattended monitoring tasks [9]. Consequently, the achievement of self-powering of sensor nodes has become an important topic in the development of aviation pipeline monitoring systems.

Vibration energy harvesting (VEH) is a novel self-powering technology that collects vibrational energy from the environment and converts it into electrical energy [10,11,12]. During flight, aircraft generate a significant amount of vibrations, which provide a stable energy source for sensor nodes [13]. By applying VEH technology, the power supply issues of sensor nodes can be effectively addressed, ensuring the long-term stable operation of WSN in aviation pipeline monitoring.

In recent years, piezoelectric materials have been extensively researched and applied in the field of VEH [14]. Piezoelectric materials possess excellent electromechanical coupling properties, enabling the conversion of mechanical vibrations into electrical energy, thus providing power support for sensor nodes [15]. By optimizing the structural design of piezoelectric materials, the efficiency of energy harvesting can be improved, meeting energy demands across different vibration frequencies. By integrating WSN with VEH technology, a self-powered WSN can be established, achieving real-time monitoring of aviation pipeline systems [16,17,18,19]. In the future, with continuous technological advancements, aviation pipeline monitoring systems will become more intelligent and automated, providing robust support for the safe operation of aircraft [20,21,22].

**Figure 1:**
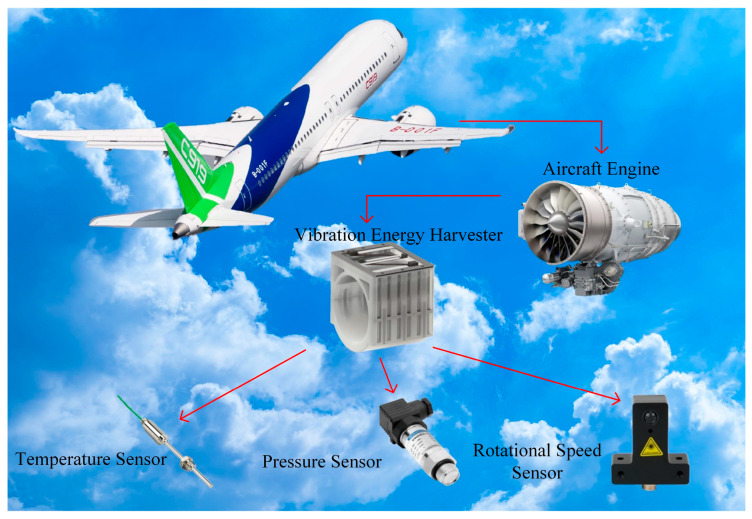
Diagram of a vibration energy harvesting system for monitoring aviation pipelines.

The VEH device can power temperature sensors, pressure sensors, and rotational speed sensors. This is crucial for the normal operation of aviation engine pipelines. Monitoring the temperature of the fuel pipeline ensures that the fuel temperature does not drop to the freezing point, preventing fuel icing, and does not pose a fire risk by rising too high [23]. Monitoring the pressure in the fuel pipeline stabilizes the air–fuel mixture ratio under working conditions, ensuring combustion efficiency and engine performance [24]. Monitoring the pressure in the lubrication oil pipeline ensures adequate lubrication for the aviation engine, preventing component wear and failure [25]. Monitoring the rotational speed of the turbine blades provides engine operation data to the pilot, ensuring flight safety. The schematic diagram of the vibration energy collection system for monitoring aircraft pipelines is shown in Figure 1.

In research on VEH, utilizing improved mechanical structures to amplify environmental vibration amplitude can enhance the efficiency of energy harvesters [26]. Liu et al. [27] designed a rainbow-type piezoelectric transducer and established an analytical model for energy conversion efficiency. They derived the optimal length-to-thickness ratio for the structure and identified the optimal structural design parameters. Their transducer achieved a conversion efficiency of 2.21%, with an optimal length ratio of 0.65. Sriramdas et al. [28] proposed a piezoelectric cantilever beam vibration energy harvester with a combined stepped thickness, capable of achieving broadband VEH in low-frequency environments. Eshtehardiha et al. [29] designed an internal resonance dual-cantilever beam energy harvester that combined a spring system with a cantilever beam system. The experimental results showed that this structure can broaden the operating frequency band of the energy harvester and improve the conversion efficiency of the VEH. Gibus et al. [30] proposed an analytical model based on the Rayleigh–Ritz method and a new two-degrees-of-freedom model. They validated these models through comparison with finite element simulations, and the experimental results demonstrated that their designed device could achieve 100 μW of output power, providing energy to power-consuming devices in WSN.

In the research on VEH systems composed of traditional piezoelectric cantilever beams, existing analyses and experiments have shown relatively efficient output performance [31]. However, owing to the slender characteristics of traditional cantilever beam structures, their application in aviation pipeline monitoring systems remains limited [32]. This paper conducts a modal analysis of three different pipeline structures in aircraft by performing simulation experiments on the number and arrangement of clamps affecting their natural frequencies. An N-shaped symmetrical vibration energy harvester was designed based on the vibration characteristics of aviation pipelines. A mounting device was also designed to install an energy harvester on the pipeline to provide power to the sensors. This setup lays the foundation for monitoring the sensor-based pipeline data. The design achieved the installation of an unconventional cantilever beam in a limited space. Theoretical analysis and finite element simulation software were used to optimize the parameters affecting the energy harvesting efficiency of the vibration energy harvester, resulting in the optimal configuration of the harvester. The feasibility of this design was validated through experiments, and a comparison of experimental results with simulation results proved its feasibility. This design holds significant practical engineering value for the structural health monitoring of aviation pipelines.

## 2. Analysis of Vibration Characteristics of Aviation Pipelines

**Figure 2:**
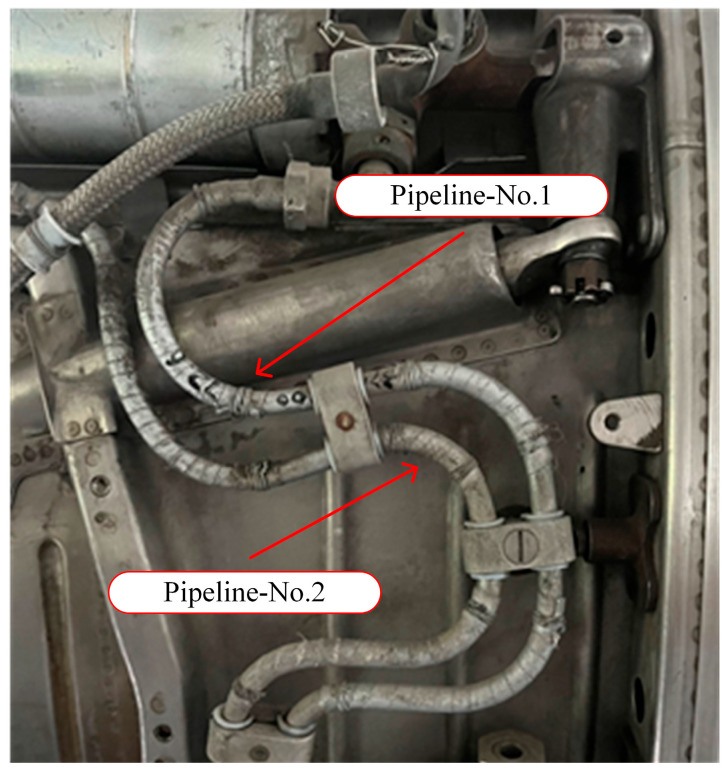
Structure of civil aircraft pipelines.

Pipelines are an essential component of aviation structures that are responsible for transporting fuel, hydraulic oil, and other fluids to ensure the normal operation of aircraft. Pipelines are typically made of materials that are resistant to high pressure and corrosion [33]. In this experiment, typical configurations of fuel and hydraulic pipelines from civil aircraft have been selected for measurement and experimentation. A physical diagram is shown in Figure 2, with a material grade of 06Cr19Ni10, density of 7930 kg/m^3^, and Poisson’s ratio of 0.3.

**Figure 3:**
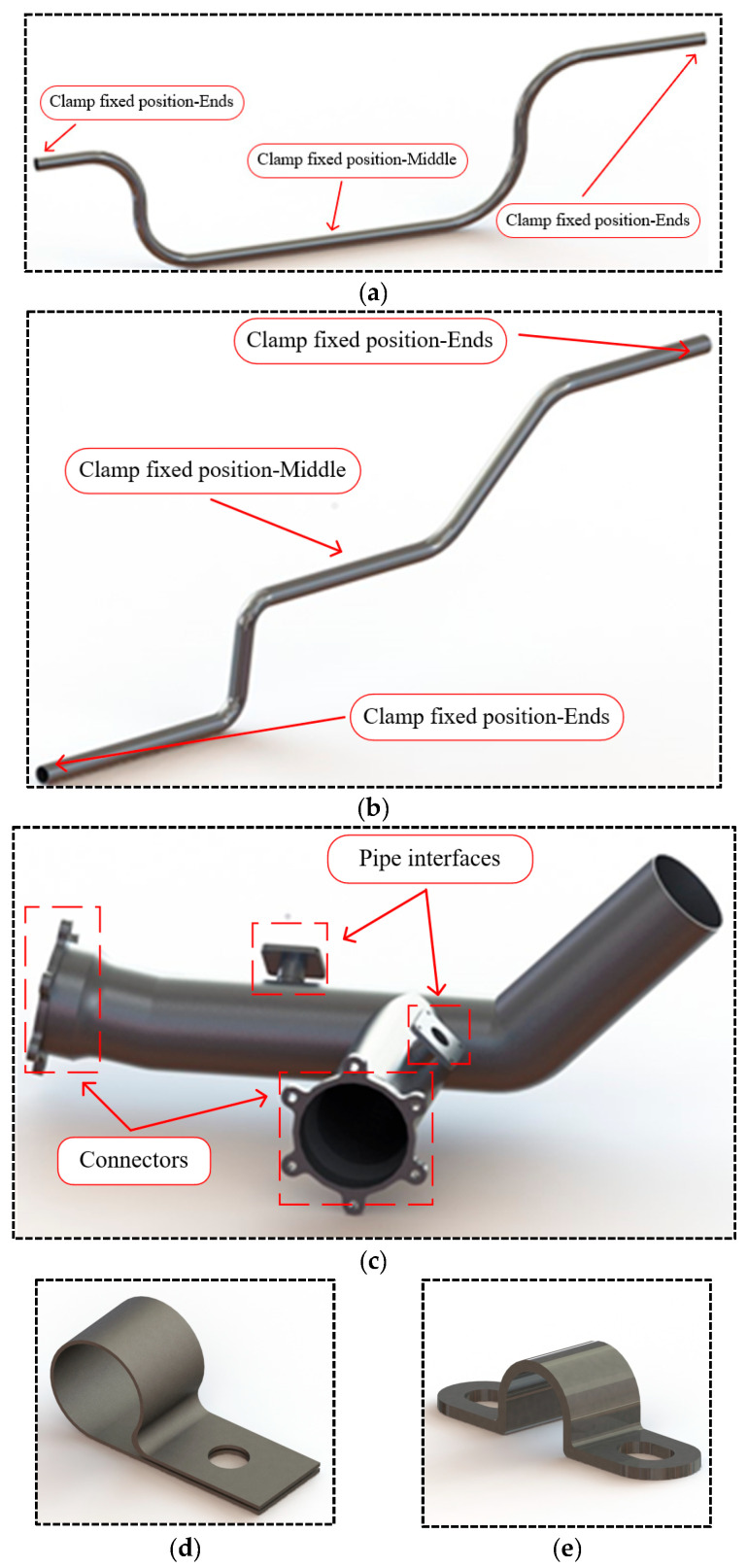
Typical pipeline structures and clamp structures in aviation equipment: (**a**) transportation pipeline 1; (**b**) transportation pipeline 2; (**c**) connection pipeline; (**d**) single-nut fixed clamp; (**e**) double-nut fixed clamp.

Modeling of the selected pipeline types from the previous text included transportation and connection pipelines. The transportation pipelines are shown in Figure 3a,b, whereas the connection pipelines are shown in Figure 3c. The transportation pipelines are connected to the connection pipelines through a docking structure. A common method of the fixation of aviation pipelines is clamp fixation. Single-nut fixed clamps are shown in Figure 3d, and double-nut fixed clamps are shown in Figure 3e.

This study investigates the vibration characteristics of the first two types of pipelines. COMSOL 6.3 simulation software was used to investigate the impact of clamp placement and number of clamps on the modal characteristics of aviation pipelines. The structural parameters of the pipelines are listed in Table 1.

The number and arrangement of clamps affect the natural frequency of the pipeline [34]. The natural frequency of the pipeline is related to the design of the vibration energy harvester. The pipeline model was simulated using simulation software to obtain the first six natural frequencies of the different pipelines under the influence of the clamp arrangements. The results are presented in Table 2.

**Figure 4:**
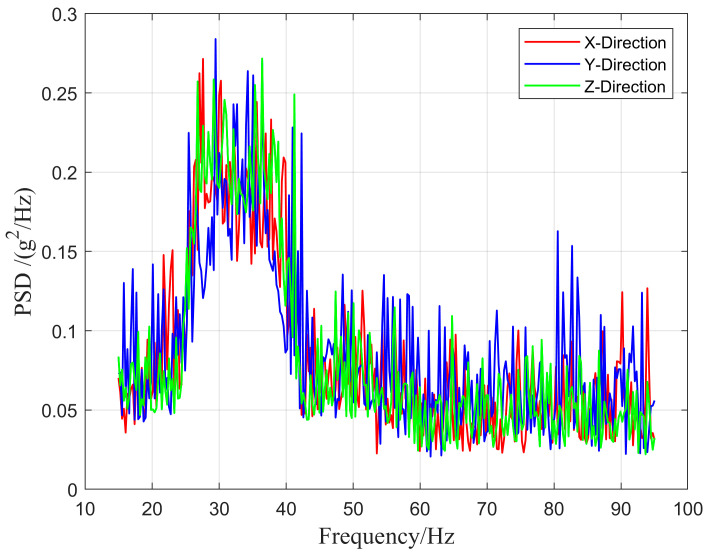
Typical load spectrum of civil aircraft pipeline.

Based on the analysis of the first six modes of the two types of transportation pipelines, it can be concluded that the natural frequencies of the pipelines are affected by different clamp placement conditions and that increasing the number of clamps increases the first six natural frequencies of the pipelines. This study focuses on pipelines fixed under single-clamp conditions and analyzes the design of vibration energy harvesters in this frequency environment. The goal is to design an energy harvesting device that can operate efficiently over a wide working frequency band in low-frequency environments. The vibration signals of the civil aircraft fuel and hydraulic pipelines were measured in the experiment, and the power spectral density (PSD) of the pipeline vibration was obtained through mathematical transformations, as shown in Figure 4.

**Figure 5:**
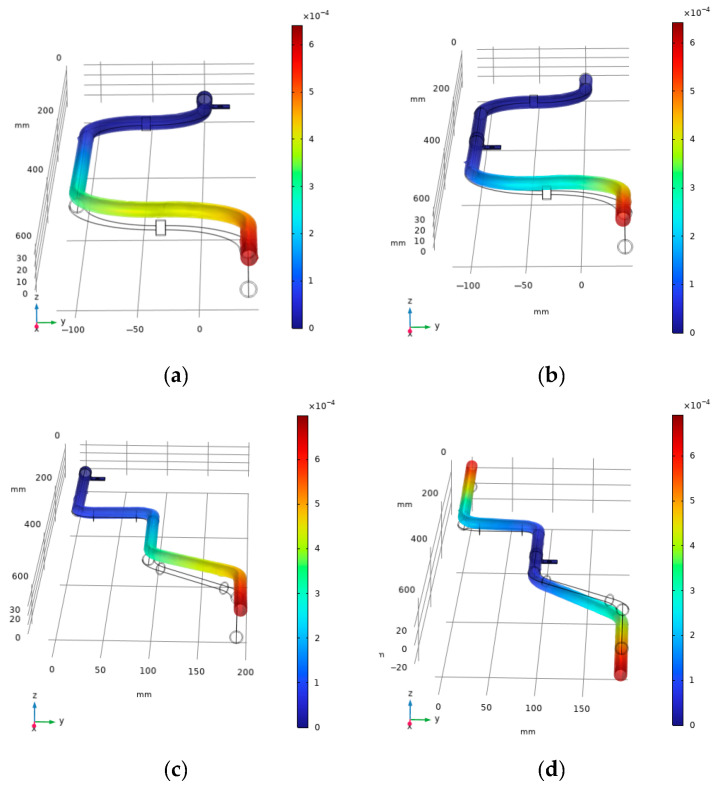
First mode shape diagrams for various types of pipelines and single-clamp fixation methods: (**a**) pipeline 1—single clamp at one end; (**b**) pipeline 1—single clamp in the middle; (**c**) pipeline 2—single clamp at one end; (**d**) pipeline 2—single clamp in the middle.

Based on the curves in Figure 4, vibrations exist in the X-, Y-, and Z-directions of the aviation pipeline. Therefore, the efficiency of the VEH device used in this experiment should be considered under multi-directional excitation. Furthermore, regarding the power distribution of vibrations, the excitation signals within the frequency range of 20–40 Hz exhibit the highest power, emphasizing the need for an experimental design to focus on the collection efficiency of the energy harvester in low-frequency environments. Regarding the placement of the vibration energy harvester, the vibration mode diagrams of the pipelines fixed under the single-clamp condition considered in this experiment were plotted, and the results are shown in Figure 5.

The first mode shape diagrams for the two types of pipelines under different fixation methods are shown in the figure. The maximum displacements for both types of pipelines occur at the ends under the current frequency. Therefore, when placing the VEH device, it is preferable to position the collector there. Moreover, by analyzing the vibration modes of aircraft pipelines, the vibration energy harvesting device can be prevented from resonating with the pipelines, thereby ensuring the operational safety of the aircraft pipeline structure.

## 3. N-Shaped Symmetrical Vibration Energy Harvester Model

To power the structural health monitoring system of aviation pipelines, this paper designs an N-shaped symmetrical vibration energy harvester. It improves the traditional piezoelectric cantilever beam’s geometric structure by designing the cantilever beam part as an N-type configuration. This configuration can be considered as a combination of three cantilever beams. The geometric structure enhances spatial utilization efficiency in static characteristics. Compared to the single slender piezoelectric cantilever beam structure, this design can be installed on narrow pipelines through matching fixtures. Furthermore, compared to the traditional piezoelectric cantilever beam, the N-type piezoelectric cantilever beam structure also has a broader operating frequency band, leading to better energy harvesting efficiency.

### 3.1. Structural Design of Energy Harvesting Device

Optimizing the structure of a cantilever beam-type vibration energy harvester can improve the efficiency of piezoelectric energy harvesting [35]. Currently, research on optimizing the piezoelectric cantilever beam structure is mainly focused on adjusting the combination of the cross-sectional shape of the piezoelectric cantilever beam and adjusting the mass of the counterweight to achieve energy extraction under composite modes [36,37,38]. However, in aviation pipeline monitoring applications, the space around the aircraft engine compartment and transmission pipelines is limited, and the placement space for VEH structures is restricted. Moreover, the actual vibration modes in these scenarios are diverse, making it challenging to efficiently place and collect energy using simple cantilever beam structures. This greatly limits the placement of energy harvesters and reduces the energy harvesting efficiency. This study proposes an N-shaped symmetrical vibration energy harvester to address the structural health monitoring of aviation pipelines.

**Figure 6:**
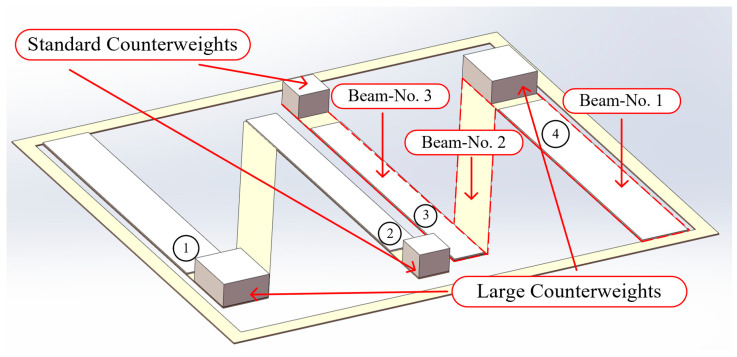
Vibration energy harvester structural diagram.

Figure 6 shows a schematic of the geometric model of the VEH device. The components of the symmetrical N-shaped piezoelectric cantilever beam vibration energy harvester include two sets of N-shaped cantilever beams, standard counterweights, large counterweights, standard piezoelectric sheets, and wide piezoelectric sheets. The fixed end of the first set of N-shaped cantilever beams was installed opposite to that of the second set of N-shaped cantilever beams. The N-shaped cantilever beams included beams No. 1, No. 2, and No. 3. Beams No. 1 and No. 3 were parallel, and the angle between beams No. 2 and No. 1 was 30°.

In the figure, the yellow part is the N-shaped cantilever beam substrate, and the white material installed on top is the piezoelectric ceramic. The width of beam No. 1 of the N-type cantilever beam is greater than the width of beam No. 2 and beam No. 3. A standard-width piezoelectric patch and a wide piezoelectric patch are installed on the substrate of the cantilever beam. Among them, the wide cantilever beam is installed on the first beam part of the N-type cantilever beam substrate, and the standard piezoelectric patch is installed on the third beam part of the N-type cantilever beam substrate.

### 3.2. The Mechanical Model of the Device

The vibration equation of a cantilever beam can be derived from the dynamic theory of a simple cantilever beam model. Under external excitation, the free end of the cantilever beam generates lateral and longitudinal displacements. In the case of no damping, the vibration equation of a cantilever beam with a uniform cross-section can be written as follows [39]:

(1) $$EI\frac{d^{4}\omega (x,t)}{dx^{4}}+\rho A\frac{d^{2}\omega (x,t)}{dt^{2}}=0$$

where *I* is the moment of inertia of the cantilever beam cross-section, *A* is the cross-sectional area of the cantilever beam, *E* is Young’s modulus of the cantilever beam, *ρ* is the material density of the cantilever beam, and ω(x,t) is the lateral displacement of a point at coordinate *x* at time *t*. Based on the fourth-order constant coefficient partial differential equation from Equation (1), ω(x,t) is solved using the method of variable separation.

(2) ω(x,t)=W(x)q(t)

**Figure 7:**
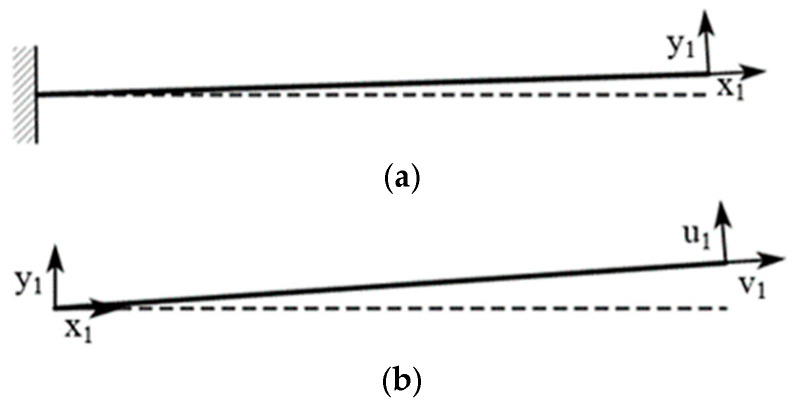
Simplified schematic diagram of displacement for a single cantilever beam: (**a**) schematic diagram of displacement for cantilever beam No. 1; (**b**) schematic diagram of displacement for cantilever beam No. 2.

Substituting the expression from Equation (2) into Equation (1), we obtain the following:

(3) $$\frac{EI}{\rho A}\frac{W^{\left(4\right)}(x)}{W(x)}=-\frac{\ddot{q}(t)}{q(t)}$$

where the left side is a function of *x*, the right side is a function of *t*, and the fractions on the left and right sides are independent of each other. Therefore, these parameters must remain constant. Equation (3) can be separated into two independent ordinary differential equations:

(4) $$\left\{\begin{array}{c} W^{\left(4\right)}(x)-s^{4}W(x)=0\\ \ddot{q}(t)+\omega q(t)=0 \end{array}\right.$$

where *s* is defined as $\frac{\rho A}{EI}\omega^{4}$; solving Equation (4) yields the following:

(5) $$\left\{\begin{array}{c} W(x)=A_{1}\cos sx+A_{2}\cosh sx+A_{3}\sin sx+A_{4}\sinh sx\\ q(t)=B_{1}\cos\omega t+B_{2}\sin\omega t \end{array}\right.$$

where the equation contains the undetermined natural frequency *ω*. By substituting the boundary conditions, *A*_1_, *A*_2_, *A*_3_, *A*_4_, *B*_1_, and *B*_2_ can be solved, and the natural frequency can be determined. Figure 7 shows a schematic of the displacement of a simplified single cantilever beam. As beam No. 1 was fixed on the substrate, its equivalent displacement schematic is shown in Figure 7a. Beam No. 2 was connected to the free end of beam No. 1, and its equivalent displacement schematic is shown in Figure 7b.

The vibration in the $u_{1}$ direction of beam No. 2 in Figure 7 can be simplified as follows:

(6) $$u(x_{i},t)=U(x_{i})q_{i}(t)$$

According to Equations (4) and (6), solving the differential equation yields the following:

(7) $$\left\{\begin{array}{c} \begin{array}{l} U(x_{i})=A_{i,1}\cos s_{i}x_{i}+A_{i,2}\cosh s_{i}x_{i}+\\ A_{i,3}\sin s_{i}x_{i}+A_{i,4}\sinh s_{i}x_{i} \end{array}\\ q_{i}(t)=B_{i,1}\cos\omega_{i}t+B_{i,2}\sin\omega_{i}t \end{array}\right.$$

The boundary conditions of beam No. 2 at $x_{i}=0$ must be solved according to Equation (5). Combining this with the boundary conditions at $x_{i}=l_{1}$ (the right end in Figure 7b), the constant terms can be solved for, or their ratios can be determined. This process yields various natural frequencies. For a simple cantilever beam model, the boundary conditions can be applied to solve for *A_1_*, *A_2_*, *A_3_*, and *A_4_*, and *B_1_* and *B_2_*, and their natural frequencies can be determined. Similarly, by connecting cantilever beams with varying cross-sectional widths, it is possible to include the natural frequencies of more vibration energy harvesters within the vibration frequency range, thereby expanding the operating frequency band of the VEH device.

## 4. Energy Harvester Simulation and Parameter Optimization

Using COMSOL 6.3 Multiphysics software to construct the grid and perform finite element simulation of the model in this design, the experiment explored the effects of counterweight mass, external load, structural dimensions, and external excitation on the power generation performance of the N-shaped symmetrical vibration energy harvester.

### 4.1. Finite Element Modeling

**Figure 8:**
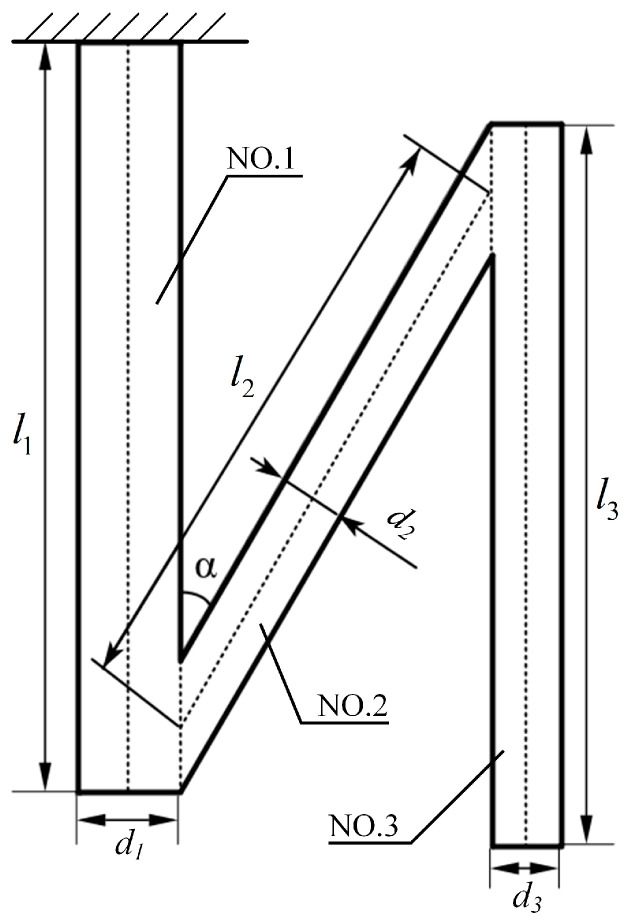
N-shaped cantilever beam geometric structure diagram.

Before conducting finite element modeling, the geometric parameters of the N-shaped cantilever beam must be determined. Figure 8 shows a simplified geometric structure diagram of a single N-shaped cantilever beam obtained from a top-down view.

Each beam segment was numbered sequentially starting from the fixed end of the cantilever beam. As shown in Figure 8, a single N-shaped cantilever beam consisted of three beam segments. Beams No. 1 and No. 3 were parallel to each other, and the angle between beams No. 1 and No. 2 was *α*. In this experiment, the substrate material of the cantilever beam is C17200 beryllium bronze, the piezoelectric material is PZT-5H, and the counterweight material is structural steel. Figure 8 defines the dimensional parameters of each beam, and the numerical values of the dimensional and material parameters are listed in Table 3.

**Figure 9:**
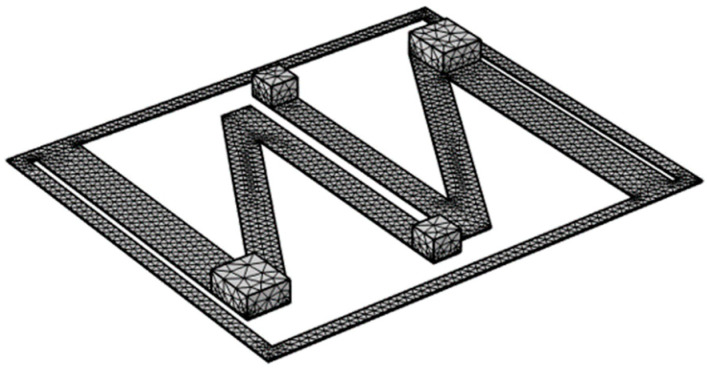
Finite element mesh generation of the vibration energy harvester.

N-shaped cantilever beams were constructed according to the parameter values defined in Table 3. Counterweights were attached to the free ends of beams No. 1 and No. 3. Based on the dimensional parameters in Table 3, it is evident that the counterweights of beams No. 1 and No. 3 are different in size. A large counterweight was attached to beam No. 1, whereas a standard counterweight was attached to beam No. 3. The counterweights were composed of structural steel with a density of 7850 kg/m^3^. The dimensions of the large counterweight were 6 mm × 7 mm × 3 mm, and those of the standard counterweight were 4 mm × 4 mm × 3 mm. Similarly, the dimensions of the piezoelectric sheets attached to different beam sections also vary, being 35 mm × 6 mm × 0.2 mm and 35 mm × 4 mm × 0.2 mm, respectively. The model was meshed in simulation software using triangular elements, and the resulting mesh is shown in Figure 9.

### 4.2. Modal Analysis of the Vibration Energy Harvester

**Figure 10:**
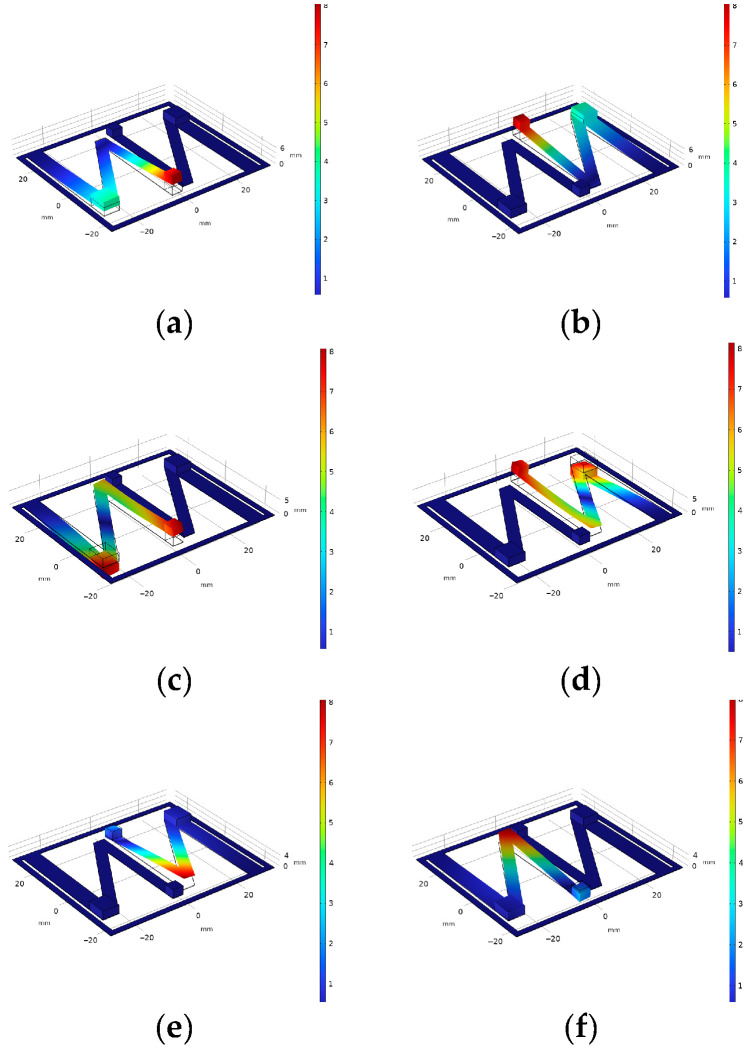
The first six modal shapes of the vibration energy harvester: (**a**) the first modal shape; (**b**) the second modal shape; (**c**) the third modal shape; (**d**) the fourth modal shape; (**e**) the fifth modal shape; (**f**) the sixth modal shape.

According to the constructed vibration energy harvester model, an analysis was conducted using finite element simulation software to extract the first six mode shapes of the model. The simulation results are shown in Figure 10.

According to the modal simulation results, the two symmetrically arranged N-shaped cantilevers had similar modal shapes. The characteristic frequencies corresponding to the first and second modes are 14.43 Hz and 15.44 Hz, respectively. For the third and fourth modes, the characteristic frequencies are 30.75 Hz and 31.74 Hz, while for the fifth and sixth modes they are 43.79 Hz and 44.81 Hz, respectively. The results exhibited symmetric characteristics. The free ends of beams No. 1 and No. 3 experienced the largest displacements. Because the model material is linearly elastic, according to Hooke’s law, maximum stress exists on these two segments of the beam. Therefore, placing piezoelectric sheets on the first and third cantilevers can improve power generation performance and output power.

Compared with the traditional piezoelectric cantilever structure, the energy harvesting results of this experiment exhibit better spatial utilization efficiency, making them more suitable for placement within aviation pipeline structures. To validate the energy harvesting efficiency, a comparative experiment was conducted between the designed single N-shaped piezoelectric cantilever structure and a simple cantilever structure. The comparison focuses on the voltage characteristics under an external load of 1 MΩ and an external excitation acceleration of 1.0 g. In the experiment, the structural dimensions of the simple piezoelectric cantilever were identical to beam No. 1 of an N-shaped piezoelectric cantilever structure. The selection and arrangement of the piezoelectric material, as well as the placement of the weight blocks, are the same as the first beam. Figure 10 shows the output voltage–frequency relationship of the two piezoelectric cantilever beam structures obtained by summing the potential differences between the upper and lower surfaces of the piezoelectric material using simulation software under an external excitation frequency range of 0–80 Hz.

**Figure 11:**
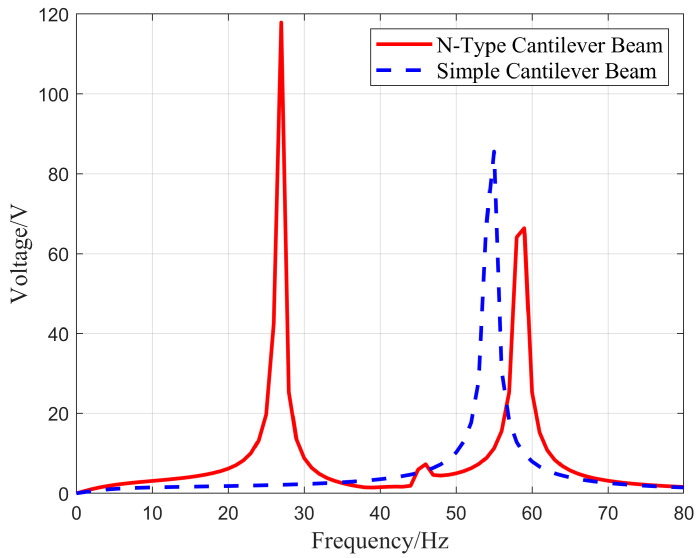
The output voltage–frequency relationship of two piezoelectric cantilever structures.

From Figure 11, it is evident that the voltage–frequency curve of the N-shaped piezoelectric cantilever exhibited two distinct voltage peaks. These peaks occur at 117.9 V at a frequency of 27 Hz, 3.6 V at a frequency of 48 Hz, and 66.3 V at a frequency of 59 Hz. In contrast, the curve for the simple piezoelectric cantilever structure shows only one voltage peak at 85.5 V, corresponding to a vibration frequency of 55 Hz. This indicates that the N-shaped piezoelectric cantilever can achieve energy harvesting in multi-modal environments at low frequencies with a broader operating frequency range. It can efficiently harvest vibrational energy in two modes, making it suitable for complex excitation environments in aviation pipeline structures. Compared to a simple cantilever, its voltage characteristics are superior, resulting in better energy harvesting efficiency. Considering the vibration characteristics of aviation pipelines, this experimental design can efficiently harvest energy during pipeline vibrations under single-nut clamp conditions.

**Figure 12:**
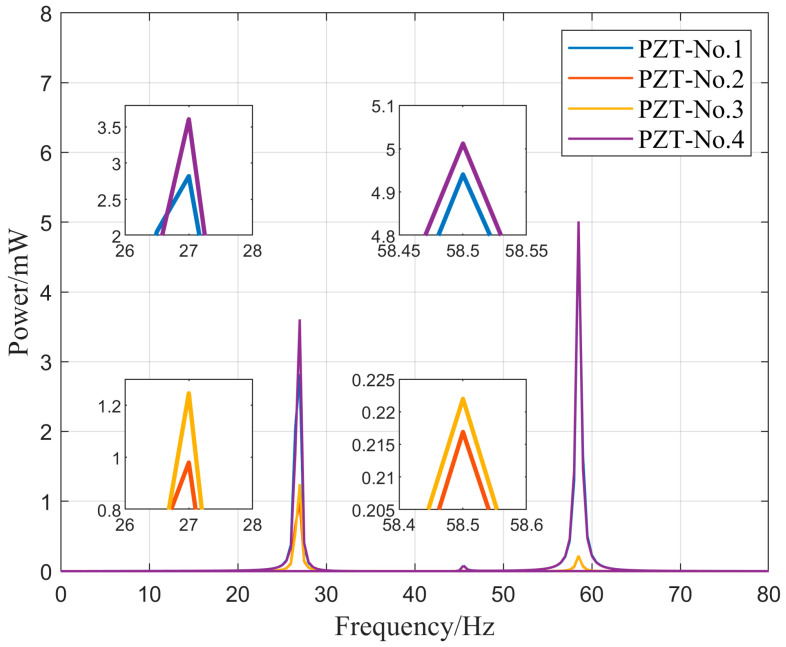
The output power–frequency relationship of four piezoelectric elements.

We numbered the piezoelectric materials in Figure 5 sequentially from 1 to 4 and then simulated their output characteristics. Figure 11 and Figure 12 show the output power–frequency curves of each piezoelectric patch in the symmetrical N-type piezoelectric cantilever beam used in this design. This set of simulations compares the output power of piezoelectric patches 1 through 4 under external excitation frequencies ranging from 0 to 80 Hz, while keeping the external load and excitation unchanged.

Based on the dimensions and placement of the piezoelectric materials, it can be observed that piezoelectric sheets No. 1 and No. 4 exhibit similar output power, while piezoelectric sheets No. 2 and No. 3 also demonstrate a similar pattern. From Figure 12, it is evident that in this design, owing to the symmetric placement of the two sets of N-shaped cantilevers with their fixed ends facing each other, they exhibit different output powers at the same external excitation frequency. Specifically, at external vibration frequencies of 27 Hz and 58.5 Hz, piezoelectric sheet No. 4 has the maximum output power, measuring 3.6 mW and 5 mW, respectively.

### 4.3. Parameter Optimization for Vibration Energy Harvester

To investigate the impact of the parameters of the combined N-shaped piezoelectric cantilever on its power generation efficiency, the relationship between the external excitation acceleration, mass of the counterweight, combination methods, length of piezoelectric sheets, and placement of piezoelectric sheets with the output power of the energy harvester was analyzed. The simulation experiment was conducted using COMSOL Multiphysics software. According to [40], the damping type set in the simulation software is the isotropic loss factor, denoted as $\eta_{s}$, with a value of 0.001.

The constructed N-shaped piezoelectric cantilever structure in the experiment comprised beams of different widths. Therefore, the dimensions of the counterweights placed at the free ends of each component beam were also different. Because all materials used were structural steel, adjusting their height was sufficient to regulate the mass of the counterweight. The energy harvesting device designed in this experiment was a multi-beam combination resonant system, allowing for various combinations of the two sizes of counterweight to achieve maximum power output. The combination methods of the counterweight are listed in Table 4.

**Figure 13:**
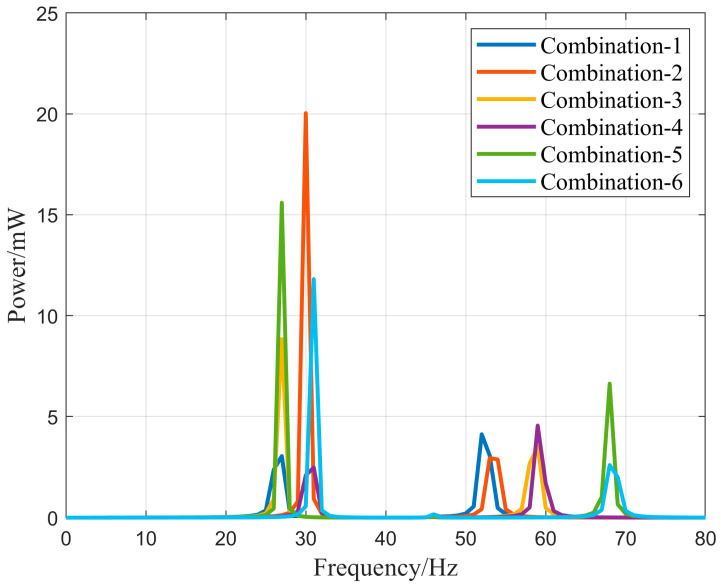
The output power–frequency relationship of different counterweight combinations.

The output power–frequency relationship of six combinations of energy harvesters under the condition of external excitation frequency from 0 to 80 Hz, acceleration of 1.0 g, and an external load impedance of 1 MΩ is compared. As shown in Figure 13, all six combinations exhibit two output power peaks in the 0–80 Hz range. Among them, Combination 2 had the highest output power in the low-frequency vibration range compared to the other combinations, indicating good performance in a low-frequency environment. Combination 5 showed a relatively high output power in high-frequency vibration environments and maintained efficient power generation in low-frequency vibration environments. The curves demonstrate that by improving the combination of the N-shaped cantilever beams, the operating frequency band of the vibration energy harvester can be adjusted, allowing it to operate in the desired vibration environment. An appropriate combination of counterweights can be selected based on the frequency range of the peak in the curve to efficiently harvest energy from environmental vibrations.

**Figure 14:**
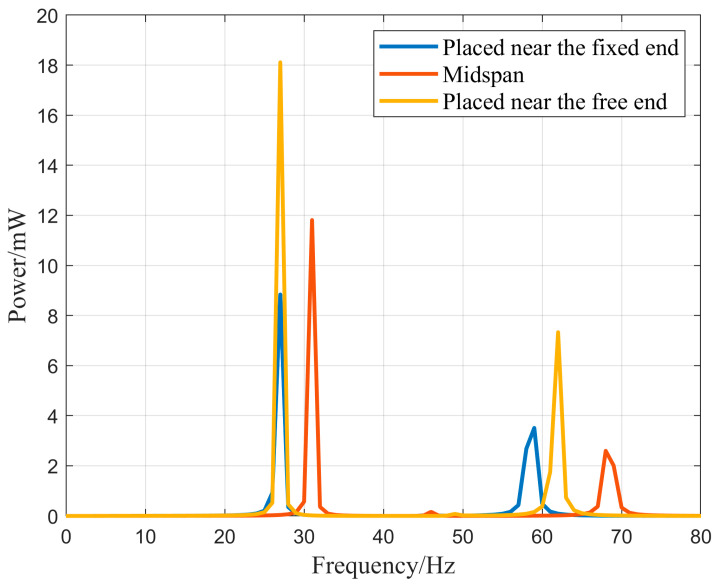
The output power–frequency relationship of different PZT placement positions.

According to the vibration mode diagram of the N-shaped energy harvester, it can be observed that the displacement distribution of the N-shaped cantilever beam is different from that of the traditional cantilever beam. Therefore, the experiment investigated the effect of the installation position of the piezoelectric sheet on the power generation efficiency of the N-shaped cantilever beam. The experiment selected the installation positions of the piezoelectric sheets near the free end, middle section, and fixed end to obtain the power–frequency relationship of the energy harvester. Under the conditions of an external excitation acceleration of 1.0 g and an external load of 1 MΩ, the output power–frequency relationship of the energy harvester in the vibration frequency range of 0–80 Hz was simulated. The simulation results are shown in Figure 14. Based on the power generation–frequency curve of the energy harvester, it can be seen that the method of fixing the piezoelectric sheet to the free end of the cantilever beam has the best power generation output. Its peak output power is located at vibration frequencies of 27 Hz and 62 Hz, with powers of 18.1 mW and 7.3 mW, respectively. The power generation efficiencies of the other two fixing methods were poor, and their operating frequencies changed.

**Figure 15:**
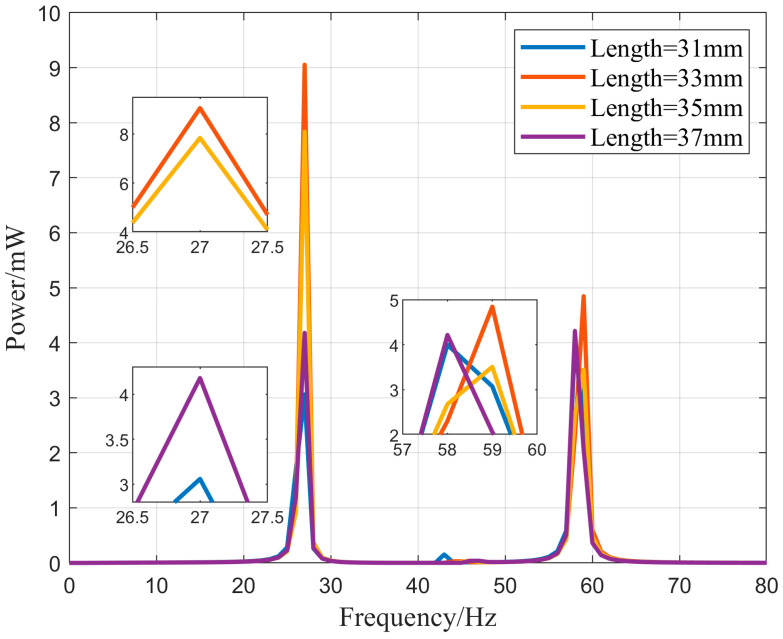
Different piezoelectric material lengths output power–frequency relationship.

To investigate the effect of the piezoelectric sheet length on the power generation output of the vibration energy harvester, PZT-5H was selected under the conditions of an external excitation acceleration of 1.0 g and an external load of 1 MΩ. The power generation output power–frequency relationship of the vibration energy harvester with the piezoelectric sheets placed near the fixed end is simulated for vibration frequencies in the range of 0–80 Hz. The output power is shown in Figure 15 for piezoelectric plate lengths of 31, 33, 35, and 37 mm. From the graph, it can be observed that when the length of the piezoelectric sheet was 33 mm, an output power of 9.1 mW was obtained at a vibration frequency of 27 Hz. At this point, the vibration energy harvester exhibited the best energy harvesting efficiency. Shortening or lengthening the piezoelectric plate resulted in a decrease in the output power. The power generation of the 37 mm long piezoelectric sheet indicates that, in this design of the vibration energy collection structure, solely relying on lengthening the piezoelectric material cannot enhance the power generation efficiency. Instead, it has a detrimental effect on energy harvesting efficiency.

**Figure 16:**
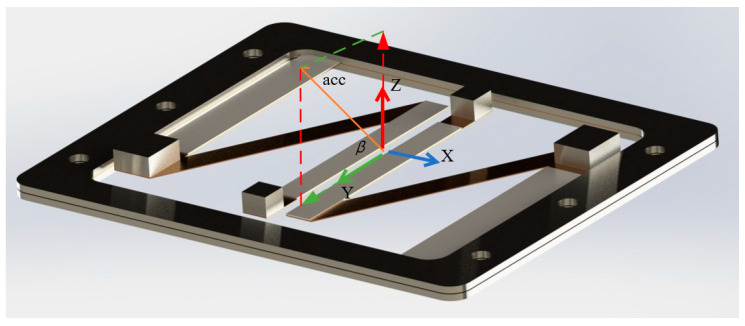
Decomposition diagram of external excitation acceleration.

Considering the multi-directional vibrations in aviation pipeline structures and to meet practical design requirements, the influence of the external excitation acceleration direction on the energy harvesting efficiency of the N-shaped cantilever beam energy harvesting structure was explored. By adjusting the angle of the exciter, external excitation acceleration at different angles can be generated. The external excitation acceleration, denoted as “acc”, is decomposed into the *Y*-axis and *Z*-axis directions based on angle *β*. The output power of the vibration energy harvester was compared under external excitation accelerations at angles of 30°, 60°, and 90° with respect to the X–Y plane. Figure 16 illustrates the decomposition of the external excitation acceleration in the Z- and Y-directions.

**Figure 17:**
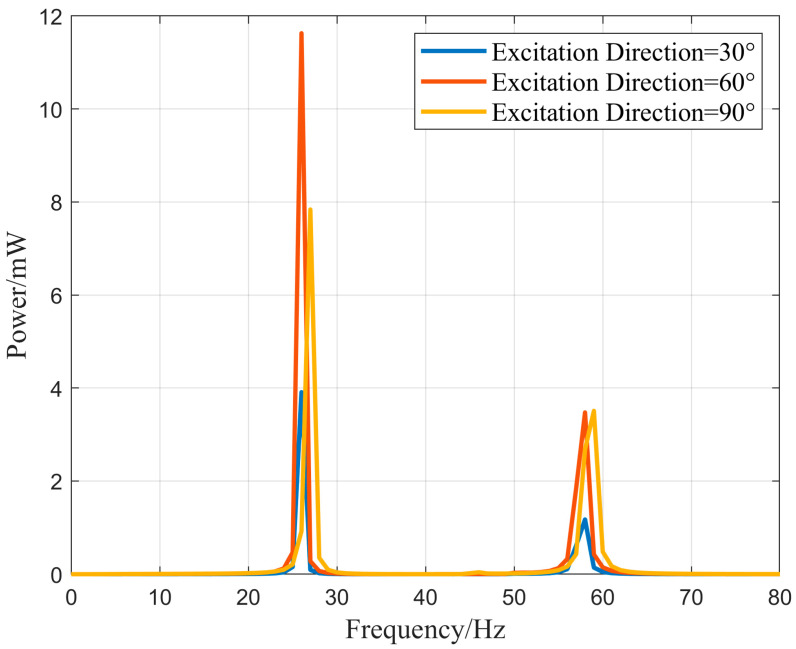
Effects of output power–frequency relationship under different directions of external excitation acceleration.

Figure 17 shows the output power of the vibration energy harvester under different directions of external excitation acceleration. According to the graph, when the external excitation acceleration reaches a 60° angle with the X–Y plane, the vibration energy harvester achieves the maximum output power. At a vibration frequency of 26 Hz, it can generate an output power of 11.6 mW, which is higher than the 7.8 mW output power at 27 Hz under vertical excitation. The simulation results demonstrate that the energy harvesting structure in this design can efficiently collect energy from multi-directional vibrations, which is beneficial for placement in different positions of aviation pipelines or for collecting vibration energy from different excitation directions. Compared with traditional piezoelectric cantilever beam structures, this design is more practical for engineering applications.

## 5. Experiment Results

**Figure 18:**
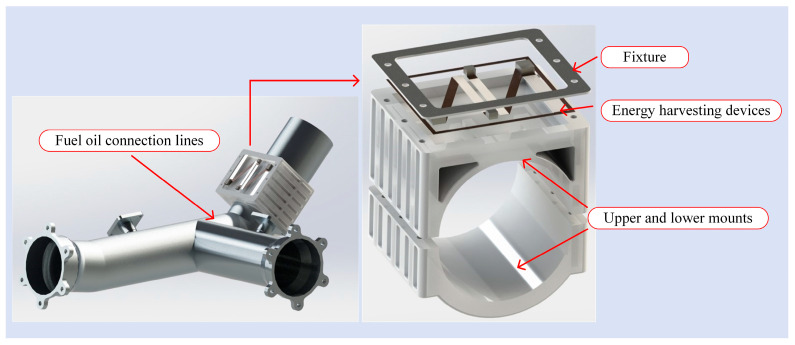
Pipeline symbiotic VEH device.

Based on the structure and characteristics of aviation pipelines, an energy harvesting device with upper and lower mounting seats for pipelines was designed. The structure, as shown in Figure 18, is installed on fuel delivery pipelines to absorb vibration energy and convert it into electrical energy.

**Figure 19:**
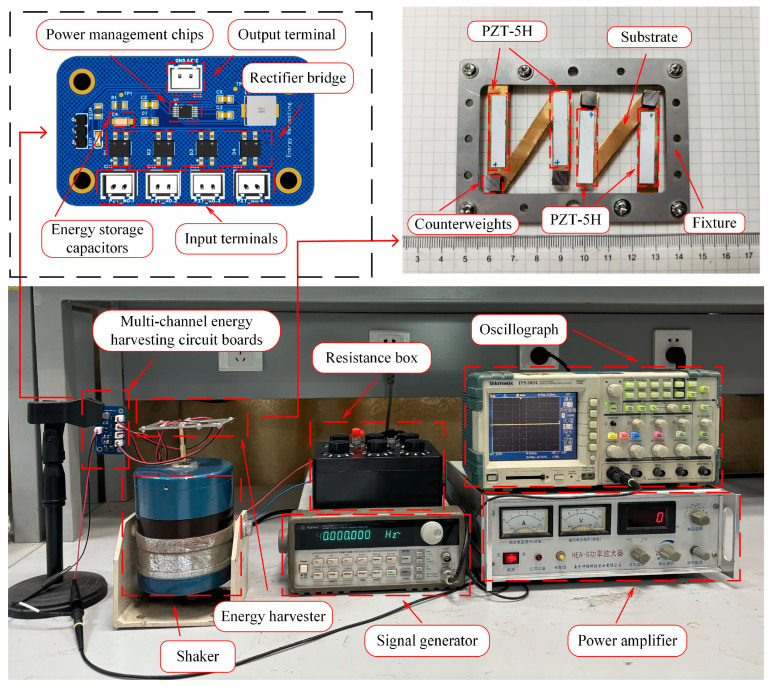
The experimental platform and the energy harvesting circuit.

The structure shown in Figure 18 is a symbiotic energy harvesting structure for pipelines consisting of upper and lower mounting seats and a top clamp. A clamp was used to fix the symmetrical N-shaped cantilever beam for energy harvesting. The upper and lower mounting seats are designed with slots to satisfy the heat dissipation requirements of the pipelines while adhering to the principle of structural light weight. For the VEH component, the N-shaped cantilever beam substrate was fixed using a clamp, and PZT-5H and a counterweight were bonded using an adhesive. The experimental equipment and energy harvesting circuit design are shown in Figure 19.

**Figure 20:**
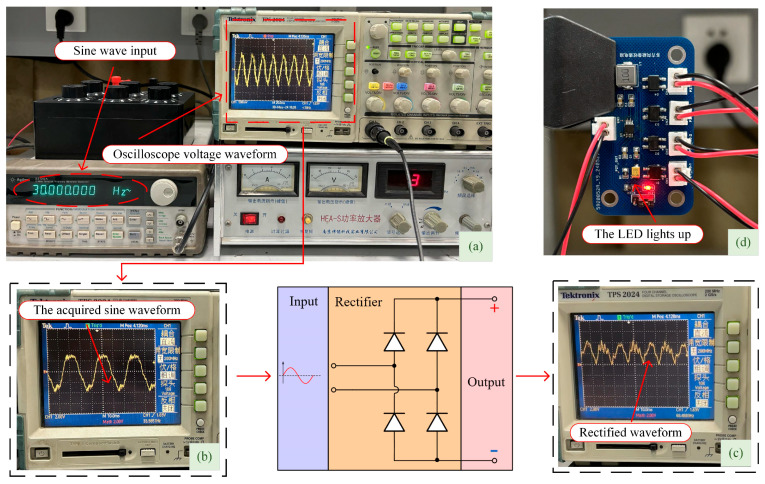
The output waveform and result plot of the vibration experiment: (**a**) experimental results; (**b**) oscilloscope output waveform; (**c**) rectified waveform; (**d**) working indicator light on.

The figure shows an experimental platform consisting of an oscilloscope (TPS2024 Tektronix, USA), power amplifier (HEA-S Yingpu, China), signal generator (33120A Agilent, USA), resistor box, and shaker. The energy harvesting circuit includes a rectification section, energy storage section, power management section, and electrical energy output section. Each energy harvesting circuit board has four external input interfaces and one output interface, capable of outputting 3.3 V voltage, with an onboard LED light that illuminates when the circuit is active to indicate operation. When the signal generator outputs a sine wave with a frequency of 30 Hz and an amplitude of 3 V, the waveform collected by the oscilloscope from the piezoelectric ceramic is displayed, as shown in Figure 20.

Figure 20a,b show the output voltage waveforms of a single piezoelectric sheet, which match the characteristics of the waveform output by the signal generator. Figure 20c shows the output voltage waveform of the piezoelectric sheet after passing through the full-wave rectifier bridge. It was observed that the negative half-cycle voltage of the piezoelectric sheet output reverses after full-wave rectification, which can be stored using a storage capacitor and controlled for output through the power management chip. Figure 20d illustrates the completion of energy harvesting in the energy collection circuit, resulting in the illumination of the onboard LED indicator light.

**Figure 21:**
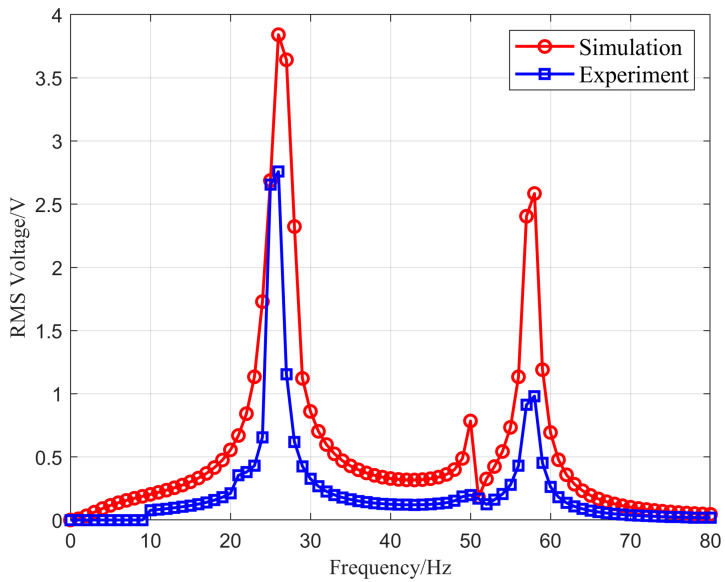
Experimental and simulation values comparison of the RMS output voltage.

To investigate the consistency between experimental and simulation results, a signal generator was used to perform frequency sweeping with a frequency step of 1 Hz. When the external excitation direction is vertical and the external impedance is 10 MΩ, the root-mean-square (RMS) voltage output curve of the energy harvesting structure with counterweight combination 2 under vibration excitation frequencies ranging from 0 to 80 Hz is shown in Figure 21.

Based on the comparison between experimental and simulation results, it can be seen that the frequency characteristics of the VEH device designed in this study show agreement. The RMS voltage obtained from the experiments is lower than the simulation results. The simulation results exhibit two high voltage peaks and one low voltage peak, specifically 3.85 V, 2.62 V, and 0.78 V, respectively. The experimental results show two prominent voltage peaks at 2.78 V and 0.98 V. Additionally, there is an insignificant voltage peak at 49 Hz with a value of 0.24 V, which significantly deviates from the simulation value. After conducting multiple experiments, we suspect this discrepancy is related to the heat treatment methods [41] (such as annealing and tempering) of the beryllium copper substrate.

**Figure 22:**
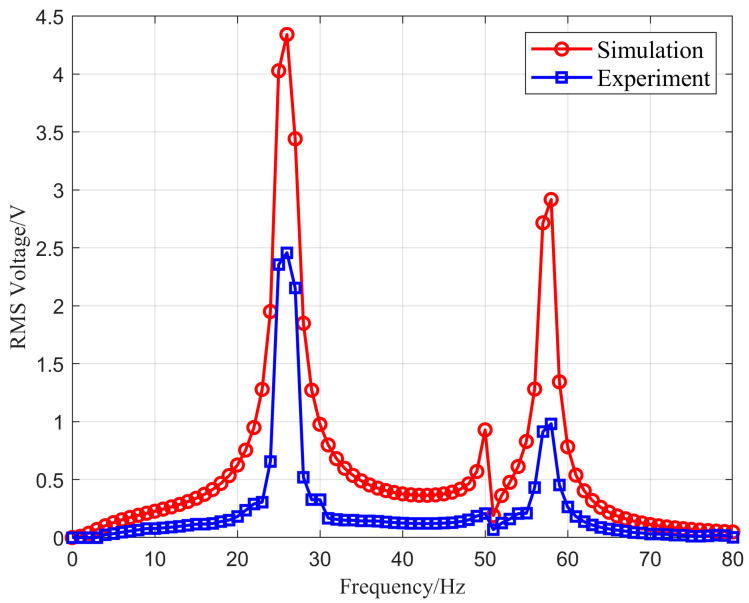
The experimental and simulation values comparison of the RMS output voltage at an excitation angle of 60°.

For multi-directional vibrations in aviation pipelines, an experiment was conducted to investigate the influence of the excitation direction on the output voltage. By adjusting the excitation direction of the exciter to provide vibration excitation at an angle of 60° for the energy harvesting device, the relationship between the experimental and simulation values is shown in Figure 22.

According to the figure, it can be seen that at an excitation angle of 60° the experimental and simulation values exhibit similar characteristics. The simulation values show two high voltage peaks of 3.48 V and 2.58 V and a low voltage peak of 0.97 V. In comparison, the experimental values display two high voltage peaks of 1.45 V and 0.98 V and a low voltage peak of 0.26 V, occurring at a vibration frequency of 49 Hz. These results indicate that the vibration energy harvesting device designed in this study is capable of capturing multi-directional vibration energy, fulfilling the functional requirements for structural health monitoring of aviation pipelines.

**Figure 23:**
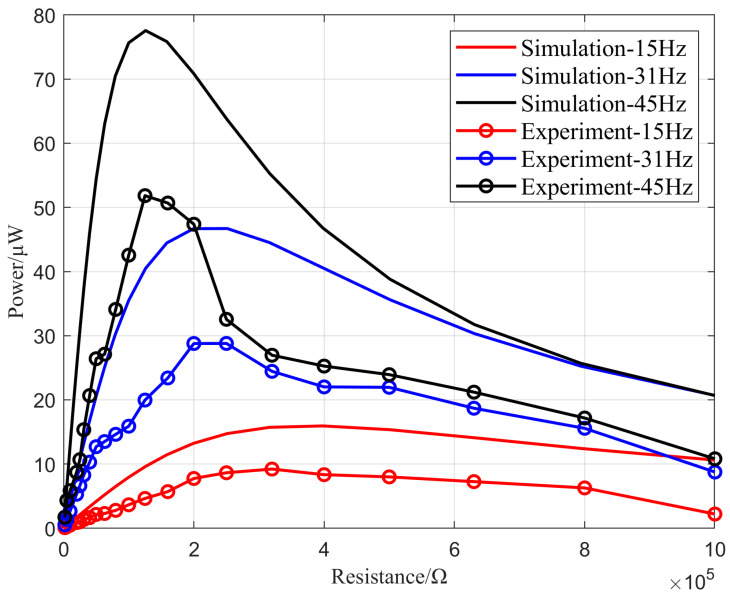
The power–load relationship of the N-shaped piezoelectric cantilever beam.

Using a resistor box for the impedance matching experiments, Figure 23 shows the power–load relationship curve of the N-type vibrating cantilever beam. To achieve higher output power from the vibration energy harvester, the first six natural frequencies of the energy harvester should be selected as the external excitation vibration frequencies. Due to the symmetrical nature of this design, three frequencies can be chosen from the first six natural frequencies of the energy harvesting device as the experimental frequencies for the power–load relationship. Specifically, 15 Hz, 31 Hz, and 45 Hz were selected as the experimental frequencies, with the external load varying in the range of 0–1 MΩ.

According to the simulation results in Figure 23, the optimal load for an external vibration frequency of 15 Hz was 316 KΩ, with an output power of 15.7 μW. At a frequency of 31 Hz, the optimal load was 19.9 KΩ, with an output power of 46.7 μW. For a frequency of 45 Hz, the optimal load was 125 KΩ, with an output power of 77.5 μW. Based on the experimental results, the range of optimal loads matched the simulation experiments, although the output power was lower than that of the simulation results. The three peak output powers were 51.8 μW at an external resistance of 125 KΩ, 28 μW at 200 KΩ, and 9.2 μW at 320 KΩ.

## 6. Conclusions

The requirement of providing energy to energy-consuming equipment in the structural health monitoring of aviation pipelines prompted this study. The vibration characteristics of aviation pipeline structures were investigated using simulation software and actual measurements to determine the effect of the number and arrangement of clamps on the vibration modes of aviation pipeline structures and vibration power spectrum diagram. Based on this, the design requirements for the vibration energy harvester were established. Considering the limited space in aviation pipeline structures and the need of high spatial utilization efficiency required for VEH structures, this design addresses the disadvantages of traditional piezoelectric cantilever beam vibration energy harvesters in terms of large space occupation. The vibration modes of this structure were theoretically analyzed, and the design parameters of the symmetrical N-shaped cantilever beam energy harvester were optimized using simulation software. The experimental validation demonstrated that the designed composite aviation pipeline structure met the practical requirements for VEH, and the final experimental results matched the simulation results, thus demonstrating significant engineering significance.

## Figures and Tables

**Table 1:** Dimensions and Structural Parameters of the Three Types of Pipelines.

Parameters	Transportation Pipeline 1	Transportation Pipeline 2	Connection Pipeline
Inner Diameter r/mm	6	6	29
Outer Diameter R/mm	7	7	30
Total Length of Pipeline L/mm	550	880	-
Bending Angle/rad	$\frac{\pi }{2}$	$\frac{\pi }{2}$/$\frac{3\pi }{4}$	$\frac{3\pi }{4}$
Radius of Curvature/mm	50/70	20/50	30

**Table 2:** The First Six Modes of Pipelines with Different Numbers and Arrangements of Clamps.

Pipeline Type	Number and Arrangement of Clamps	First Mode/Hz	Second Mode/Hz	Third Mode/Hz	Fourth Mode/Hz	Fifth Mode/Hz	Sixth Mode/Hz
Transportation Pipeline 1	Single clamp, placed on one side	24.85	26.12	149.44	156.93	334.12	368.61
	Single clamp, in the middle	69.79	73.01	125.56	130.2	362.65	394.66
	Double clamps, on both sides	147.09	158.43	365.41	394.95	594.13	602.07
	Double clamps, placed in the middle and on one side	123.06	128.98	355.28	387.1	566.38	645.33
	Triple clamps, placed in the middle and on both sides	364.08	390.95	631.21	721.01	1632.1	1811.7
Transportation Pipeline 2	Single clamp, placed on one side	23.42	24.49	101.22	101.97	320.18	334.47
	Single clamp, in the middle	66.66	68.82	135.61	144.97	354.72	378.42
	Double clamps, on both sides	115.83	279.11	338.81	405.08	520.42	762.20
	Double clamps, placed in the middle and on one side	112.63	119.86	373.53	420.21	536.84	847.74
	Triple clamps, placed in the middle and on both sides	366.62	483.33	832.17	988.61	1112.4	1222.8

**Table 3:** Cantilever Beam Model Parameters.

Description	Parameter	Value
Beam No. 1 Width (mm)	$d_{1}$	6
Beam No. 1 Length (mm)	$l_{1}$	45
Beam No. 2 Width (mm)	$d_{2}$	4
Beam No. 2 Length (mm)	$l_{2}$	37
Beam No. 3 Width (mm)	$d_{3}$	4
Beam No. 3 Length (mm)	$l_{3}$	42
Thickness (mm)	h	0.2
Beam Angle(rad)	α	$\frac{\pi }{6}$
Density (kg/m^3^)	ρ	8250
Poisson’s Ratio	*G*	0.3
Young’s Modulus (GPa)	*E*	128

**Table 4:** Combination of Counterweights in N-shaped Cantilever Beams.

Combination	$\mathrm{Height}\;\mathrm{of}\;\mathrm{Large}\;\mathrm{Counterweight}\;(h_{w1})$	$\mathrm{Height}\;\mathrm{of}\;\mathrm{Standard}\;\mathrm{Counterweight}\;(h_{w2})$
(1)	4 mm	3 mm
(2)	4 mm	2 mm
(3)	3 mm	3 mm
(4)	3 mm	2 mm
(5)	2 mm	3 mm
(6)	2 mm	2 mm

## Data Availability

The data that support the findings of this study are available from the corresponding author upon reasonable request.
